# The Forbidden Reward. The Emergence of Parent-Child Conflicts About Food Over Time and the Influence of Parents' Communication Strategies and Feeding Practices

**DOI:** 10.3389/fpubh.2020.604702

**Published:** 2021-01-18

**Authors:** Ines Spielvogel, Brigitte Naderer, Alice Binder, Jörg Matthes

**Affiliations:** ^1^Department of Communication, Advertising and Media Effects Research Group (AdME), University of Vienna, Vienna, Austria; ^2^Department of Media and Communication, Ludwig Maximilian University of Munich, Munich, Germany

**Keywords:** parent-child conflict, food, food-related mediation, parental feeding practices, panel study

## Abstract

One of the most critical arenas for conflicts between parents and their children relates to food. Although parent-child conflicts about food are a real occurrence, this form of parent-child interaction has been rarely examined. Given the special role of parents in shaping children's diet, we especially focus on the impact of parental measures. This study investigates how parental communication strategies (i.e., active vs. restrictive) and feeding practices (i.e., overt control vs. covert control) affect the emergence of parent-child conflicts about food over time. Based on previous research, we assessed overt control through parents' use of food as a reward and restriction of their children's access to specific food types. We explored the impact of our predictors on both conflicts about unhealthy and healthy food with a two-wave panel study including parents and their children (*N* = 541; children aged between 5 and 11) in Austria between fall 2018 and spring 2019. Results of two multiple linear regressions indicated that predominantly parents' use of unhealthy food as a reward is connected to both healthy and unhealthy food conflicts. Furthermore, inconsistent parental educational styles increased the respective conflict potential. Active food-related mediation and covert control did not relate to food-related conflicts about unhealthy and healthy food. Parents' increased use of overtly controlling and restrictive feeding practices might not be only counterproductive for children's diet but also for food-related parent-child interactions. Instead, a “health discourse” (i.e., active food-related mediation) might prevent food-related conflicts and foster a healthy growth in the future.

## Highlights

- Parents' use of unhealthy food as a reward triggered food conflicts with children.
- Restrictive food-related mediation predicted some food conflicts with children.
- Inconsistent parental educational styles increased the respective conflict potential.
- Covert control could not decrease the food conflict potential but also did not further it.
- Active food-related mediation did not further conflict about food.

## Introduction

Questions about nutrition can lead to some disagreements between family members (1–4), especially when children are involved (5). Most importantly, there is a great potential for the emergence of these conflicts, as children are constantly exposed to food-related cues (6–9).

From an evolutionary perspective, providing infants both shelter and food appears to be the most fundamental form of parenting (10). Since parents significantly shape the diet of their children through the provision of food, parents are credited with a pervasive influence on the eating behaviors of their children (11–13). Imitative learning is an important concept that shapes children' learning outcomes. Hence, parents forming a role model for children is a relevant aspect (14, 15) for the process of developing food preferences. Based on this, we therefore assume that communicating rules and acting as a role model with respect to what food parents provide and consume themselves shapes children's food behavior and attitudes. Indeed, this theoretical assumption has already been studied by several scholars [for a review, see Cruwys et al. (16)]. For instance, the reported intake of both healthy and unhealthy food of individuals, who define themselves as the family food preparers, are in line with the reported eating habits of the remaining family members (17). In line with this finding, other cross-sectional studies also revealed high support for the role of parents as the main influence for children's eating behavior (12, 13, 18, 19). Existing studies in the nutrition domain furthermore suggest that modeling can also have longitudinal effects, since the copying of parents' dietary behaviors is still observable after children have left their parents' homes (20).

While parents try to maintain harmony by serving food that their children like to eat, they simultaneously tend to make food decisions based on health issues (21). As healthy food options do not always align with children's inherent preferences (22), there is an increased likelihood that parent-child conflicts break out (4). This may be especially true if parents are facing a disproportionate demand for sweets and snacks of their children (3). Given the significant role of parents for their children's diet, we argue that both, how parents communicate food-related issues to their children and how parents behave with regard to feeding practices, might be relevant for the emergence of food-related conflicts (14). In family conflict situations, parents can respond in several ways: For instance, parents may counter that their child has to finish their meal for getting dessert without or with an additional explanation; Also, parents may give in so that their child stops nagging. The question therefore is, which conflicts regarding food between parents and their children arise due to the use of such different parental feeding practices and communication styles in the day-to-day interpersonal communication of a family.

Insights into the emergence of parent-child conflicts regarding food are relevant because these types of conflicts might not only negatively affect interpersonal relationships but might also lead to the development of unhealthy eating behaviors or even eating disorders in children (2, 23, 24). The present study presents a panel-investigation with *N* = 541 parents examining emerging food conflicts based on parental communication strategies (i.e., restrictive vs. active food-related mediation) and parental feeding practices (i.e., overt vs. covert control). We define a parent-child conflict about food as an interpersonal debate between parents and children about the amount of specific food products that are consumed. We also argue that conflicts about food should be discussed separately for unhealthy and healthy foods. We therefore explore the impact on both types of conflicts.

## Parental Food-Related Mediation

How parents communicate to their children regarding healthy and unhealthy eating behaviors is considered as an important social impact on children's food behavior (25, 26). However, little is known about parental mediation regarding food. Only two studies we are aware of built on established parental mediation strategies that are used by parents to prevent advertising effects in their children (27) and transferred these strategies to the context of food (26, 28). Research in the domain of public health and nutrition distinguishes two dimensions of parental mediation about food: *Restrictive food-related mediation* and *active food-related mediation*.

Restrictive food-related mediation is defined as a strategy that is used by parents who forbid their child to eat specific food and who communicate clear rules on how much their child is allowed to eat. No further explanations on why children should follow these rules and requirements are given. Active food-related mediation, in contrast, refers to parents who explain to their children what the health benefits of specific food products and the consequences of unhealthy eating behaviors are. Hence, they contextualize their rules and appeal to the child's autonomy (26, 28).

Previous research in the context of family conflict revealed certain tendencies of different communication styles that might also apply to food-related conflicts. For instance, studies found that high conversation orientation of families (i.e., a family that is open in communication) is associated with lower conflict avoidance (29). This is based on the assumption that there might be no unresolved conflicts between the family members (29). The principal of conversation orientation may also apply to the active mediation of food-related issues as it relates to parents who frequently discuss with their children about health (dis)advantages of (un)healthy foods and consequences of unhealthy eating (26, 28). Gram (30) furthermore argues that children might acknowledge their parents as “health guardians” and may accept health as an argument as communicated in active mediation, leading to less conflict potential. Other scholars suppose that an argumentative discussion may be the key for the resolution of a conflict, as in this case parents seek to resolve the disagreement of opinion by putting forward arguments (31). We thus assume that parents' use of restrictive food-related mediation may lead to the emergence of parent-child conflict about food because the sole communication of rules and requirements without allowing discussions is at the heart of a restrictive mediation style (27). In contrast, when parents explain to their child why specific food products are healthy or not, the potential for food-related conflict is reduced. As an exploratory approach, we tested our assumptions for both parent-child conflicts about unhealthy food (**H1a, H2a**) and healthy food (**H1b, H2b**). However, we presume no differences between these two types of conflicts.

### Parental Feeding Practices

While the concept of parental food-related mediation refers to how parents tend to communicate about food-related issues, parental feeding practices include actions and behaviors of parents (32, 33). This concept includes, for instance, a parent's tendency to restrict their child's access to specific food or to pressure their child to consume more food (34). Although parents appear to use a wide range of child feeding practices, most of the widespread styles involve restrictive behaviors (34). As scholars suggest that parental restriction belongs to parental feeding practices that can be detected by the child (35), we refer to parental restriction as overt control (36).

#### Overt Control and Emerging Conflicts

Since a great part of research revealed negative associations between overt control and children's diet (37–39), parent-child conflicts about food may especially emerge if parents use feeding practices that are considered as highly controlling. This is the case for the following two forms of overt control: (A) The use of food as a reward and (B) restricting children's access to specific food types (40).

#### Food as a Reward

In the context of eating practices within families, especially unhealthy eatable products are used by parents in exchange for a desired behavior. For instance, parents tend to use sweets as a reward so that their children consume other food products that are considered as healthier. They therefore propose a trade-off: Finish your broccoli then you get ice cream as dessert (41). However, in this case, the healthy food becomes a “obstacle” that has to be surmounted (30). In this light, existing studies suggest that the use of food as a reward may lead to unintended attitudinal and behavioral responses in children: While children's preference for the food that is used as a reward increases, their preference for the food that parents want to promote decreases (42). In other words, parental “food deals” (34) do not always trigger the desired effect and can even backfire. We thus assume that parent-child conflicts about food may especially emerge if parents offer food as a reward (**H3**).

#### Food Restriction

Furthermore, when parents restrict their child's access to unhealthy food, the chance for a food-related conflict may increase, because the restricted food may be particularly alluring for their child (**H4**). Several studies showed that restricting access to specific food can lead to increased preferences for and overconsumption of the restricted food in children (43–45). For instance, Fisher and Birch (43) found that children who were restricted to a jar of cookies for 5 weeks in their home environments afterwards made more requests for the cookies and ate larger portions than children who were not exposed to the restriction during this period. Again, we tested our assumptions for both parent-child conflicts about unhealthy food (**H3a, H4a**) and healthy food (**H3b, H4b**).

#### Covert Control and Emerging Conflicts

In previous research, overt control got contrasted with another overarching type of feeding practice, namely covert control (35, 38). In contrast to overt control that can be detected by the child, “covert control involves the management of a child's eating environment in a way that may not be recognized by the child and results in healthier food choices.” [(35), p. 106] When using this form of parental feeding practice, parents for instance tend to avoid visiting places in which unhealthy food is served or having unhealthy eatable products at home. We argue that the use of feeding practices that happen outside of children's awareness might decrease the potential of food-related conflicts (**H5**). In other words, rather than using restriction, it may be more effective if parents “guide” their children toward healthy food alternatives (21). This pattern may occur for both parent-child conflicts about unhealthy food (**H5a**) and healthy food (**H5b**).

### Inconsistency of Food-Related Mediation Strategies and Feeding Practices

Previous findings in the context of health and nutrition indicate that during mealtimes the used parental feeding practices often come along with argumentative strategies (31). In the context of emerging conflicts, we therefore claim that it is relevant to consider whether an inconsistent, combined use of food-related communication styles and feeding practices trigger conflict potential between parents and children to a higher extent.

Against this background, the use of food as a reward or bribe has been frequently referenced in the previous literature on parent-child conflict about food (2, 21, 24, 30, 41). We argue that parents' application of both restrictive food-related mediation and the restrictive feeding strategy of food as a reward communicate to children an inconsistent behavior. This is based on the presumption that forbidden food such as sweets suddenly turn out to be a reward in specific situations. Conflicts may therefore most likely emerge if parents do not only communicate in a restrictive manner by avoiding certain food but also use certain food (e.g., candy) as a reward.

When it comes to the combined use of active food-related mediation and food as a reward, we presume that parents' lack of consistency (36) may become even more obvious. In this case, parents would explain to their child the consequences of unhealthy eating but at the same time use unhealthy food as a reward. Hence, children may be extremely confused. We argue that, on a cognitive level, the rewarded food might be negatively associated due to argumentative discussions. Yet, on an affective level, the rewarded food might be positively associated as children's desire for the food increases when parents use food as a reward.

For both restriction of food access and covert control, we would not assume a lack of consistency due to the following reasons: First, an obvious restriction of food access just enforces the communicated rules. The uses of both active and restrictive food-related mediation in combination with the restriction of food access thus portray consistent actions. Second, covert control is assumed not to be noticed by children (35). We thus argue that no inconsistency should occur when combining this type of feeding practice with one of both parental communication styles.

**Figure 1:**
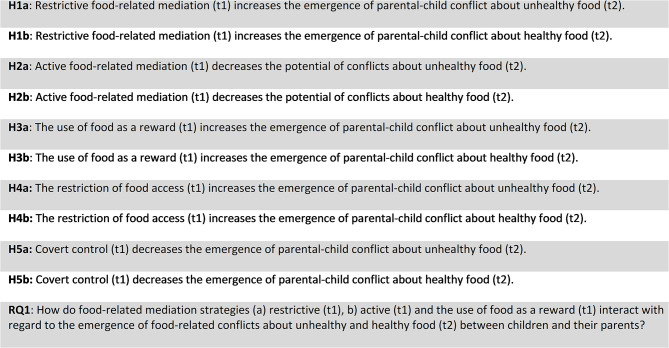
Research Assumptions Overview.

In summary, we only investigate an interaction for the feeding strategy of food as a reward and both types of parental mediation strategies (**RQ1**). Figure 1 visualizes all proposed research assumptions as an overview.

## Methods

### Design, Procedure, and Sample

We conducted a two-wave panel study in twelve primary schools in Austria. The present study was part of a bigger project but only relevant variables are reported here. The study was approved by the Ethics Committee of the University of Vienna. The first wave took place in fall 2018 followed by a second wave 6 months later. Prior to the study, parents of children between 5 and 11 years old received an information sheet, a survey, and a written consent form. The children took these documents home and returned them back to their teachers. During both waves, we collected the written consent and the filled-out surveys of the parents. For our project, we also made individual interviews with all participating children and assessed their weight and height. For our analyses, we used data of parents and children.

In the twelve participating schools, ~2,250 documents were distributed to the parents. The schools were selected based on convenience-sampling. Thus, the research-team randomly contacted different schools near Vienna and asked the headmasters if they would be interested to participate in this study. Overall, *N parents* = 829 parents between 21 and 58 years old [*n*_*parents*_ = 763; *M*_(*t*1)_ = 39.93; *SD*_(*t*1)_ = 5.74; 79.9% were female] returned documents back to the schools for the first wave (response rate: 36.84%). The educational status of the parents participating in this study was rather high (*n*_*parents*_ = 764; 51.3% obtained a university degree). Additionally, overall, *N* = 800 children between 5 and 11 years old [*n*_*children*_ = 799; *M*_(*t*1)_ = 7.80; *SD*_(*t*1)_ = 1.24; *n*_*children*_ = 796; 53.6% were female] participated in the first wave of the study. Children showed levels of Body Mass Index (BMI) between 10.97 and 30.20 (*n children* = 796; *M*_(*t*1)_ = 16.66; *SD*_(*t*1)_ = 2.61). In the present study, we excluded cases due to missing matches between the participating parent and the participating child (*n*_*parents*_ = 11) or because of incomplete parental questionnaire replies (*n*_*parents*_ = 40), leading to a sample of *N*_*parents*_ = 778 for the first response-wave. A total of 69.54% of these parents also responded in the second wave. Our final sample consisted of *N*_*parents*_ = 541 between 26 and 57 years old [*n*_*parents*_ = 525; *M*_(*t*2)_ = 40.61; *SD*_(*t*2)_ = 5.50; *n*_*parents*_ = 526; 53.2% obtained a university degree] of whom 81.6% were female (*n*_*parents*_ = 539).

### Measures

#### Independent Variables

Our measures for parental food-related mediation were based on existing research (26) and differentiated between *active food-related mediation* (three items: e.g., “I explain to my child while eating, what products are healthy.”) and *restrictive food-related mediation* (three items: e.g., “I forbid my child from eating certain products.”). Response options for the employed measures ranged from “1 = never” to “7 = very often” [active: *n*_*parents*_ = 537; α_(*t*1)_ = 0.86; *M*_(*t*1)_ = 5.55; *SD*_(*t*1)_ = 1.43; restrictive: *n*_*parents*_ = 539; α_(*t*1)_ = 0.78; *M*_(*t*1)_ = 3.95; *SD*_(*t*1)_ = 1.71].

We also asked participants for their intensity of *overt control* as a feeding strategy. We assessed their *restriction of food access* [two items: e.g., “I have to be sure that my child does not eat too many high-fat foods”; *n*_*parents*_ = 534; α_(*t*1)_ = 0.87; *M*_(*t*1)_ = 4.41; *SD*_(*t*1)_ = 1.88] and their *use of food as a reward* [two items: e.g., “I offer my child her favorite foods in exchange for good behavior”; *n* = 535; α_(*t*1)_ = 0.72; *M*_(*t*1)_ = 2.02; *SD*_(*t*1)_ = 1.33] (46). Both concepts were measured on a scale ranging from “1 = low agreement” to “7 = high agreement.”

Moreover, we assessed parents' *covert control* (35) with five items [e.g., “How often do you avoid buying sweets and crisps and bringing them into the house? “1 = never” to “7 = very often”; *n*_*parents*_ = 537; α_(*t*1)_ = 0.84; *M*_(*t*1)_ = 3.68; *SD*_(*t*1)_ = 1.56].

#### Dependent Variable

Our dependent variable was measured in both waves. We assessed the parent-child conflict about food by asking parents how often they have conflicts with their children with regard to six food categories ranging from “1 = never” to “7 = several times a day.” We entered these items into an explorative factor analysis with oblique rotation and obtained two factors, explaining 68.40% of the variance at the second wave (first wave: 63.98%; for details on the analysis results, see Table 1). The correlation between the factors for the second wave was *r*(*N*_*parents*_ = 538) = 0.45, *p* < .001 [first wave: *r*(*N*_*parents*_ = 535) = 0.36, *p* < 0.001].

**Table 1:** Factor analysis.

	**Component loadings**		***M***	***SD***
**How often do you have a conflict with your child…**				
…Because you invite your child to eat fewer sweets?	0.69		2.32	1.41
…Because you invite your child to eat fewer salty snacks (e.g., potato chips)?	0.83		1.70	1.07
…Because you invite your child to drink fewer soft drinks (e.g., coke)?	0.86		1.41	0.98
*Parent-child conflict about unhealthy food scale* (eigenvalue = 1.14, α = 0.71)			1.82	0.93
…Because you invite your child to eat more fruits?		0.93	2.20	1.64
…Because you invite your child to eat more vegetables?		0.91	2.65	1.76
…Because you invite your child to drink more water?		0.64	2.10	1.69
*Parent-child conflict about healthy food scale* (eigenvalue = 2.96, α = 0.79)			2.32	1.41

*N = 527 (14 missing values); response options: “1 = never” to “7 = several times a day”; reports represent values from the second wave*.

Based on the results of the factor analysis, we created a mean index for *parent-child conflict about unhealthy food* [*n*_*parents*_ = 536; α_(*t*1)_ =.64; *M*_(*t*1)_ = 1.75; *SD*_(*t*1)_ = 0.84; *n*_*parents*_ = 538; α_(*t*2)_ = 0.71; *M*_(*t*2)_ = 1.82; *SD*_(*t*2)_ = 0.93] which included three items related to unhealthy food categories and a mean index for *parent-child conflict about healthy food* [*n*_*parents*_ = 537; α_(*t*1)_ =.78; *M*_(*t*1)_ = 2.16; *SD*_(*t*1)_ = 1.33; *n*_*parents*_ = 539; α_(*t*2)_ = 0.79; *M*_(*t*2)_ = 2.32; *SD*_(*t*2)_ = 1.41] which included three items related to healthy food categories (for details on the measures, see Table 1).

#### Control Variables

As controls, we introduced children's age, gender, and the standard deviation score of Body Mass Index (zBMI) of the first wave. The socio-demographic characteristics of the children of our parent sample are composed as follows: approximately half of the children were female (48.3%; *n*_*children*_ = 528) and their age ranged between 5 and 11 years (*n*_*children*_ = 529; *M* = 7.78; *SD* = 1.20). In order to be able to assess children's BMI, we measured children's weight and height (*n*_*children*_ = 528; *M* = 16.46; *SD* = 2.40). Afterwards, zBMI was computed to adjust for age and gender in line with WHO (47) standards (48). Of our sample (*n*_*children*_ = 525), 19.6% children are characterized as overweight or obese since their zBMI scores were above the cut-offs of normal weight^1^.

Inspired by Austin et al. (49) we also controlled for overall *familiar acceptance of healthy food* [measured with three items; e.g., “If healthy meals are served my family enjoys it; *n*_*parents*_ = 538; α_(*t*1)_ = 0.92; *M*_(*t*1)_ = 5.07; *SD*_(*t*1)_ = 1.29: 7-point scale].

### Data Analysis

We ran two multiple linear regression models in SPSS only varying the dependent variable (i.e., conflict about *unhealthy food*; conflict about *healthy food*). In both models, we entered our variables block-wise. As a first step, we controlled for the autoregressive effect. The second step of the analyses included all hypothesized predictors, namely parental food-related mediation (active, restrictive), overt (restriction of food access, food as a reward), and covert control. Furthermore, we simultaneously inserted our control variables: children's age, children's gender, children's zBMI scores, as well as the acceptance of healthy food within the family as an indicator for established eating habits. As a last step, we inserted two interaction terms in our regression model (active food-related mediation ^*^ use of food as a reward; restrictive food-related mediation ^*^ use of food as a reward). All variables of the interaction terms were mean-centered.

## Results

### Bivariate Correlations

Before we calculated the described regression analysis we ran bivariate correlations for all variables in our model. With regard to conflict about unhealthy food at time 2, we found significant relationships with conflict about unhealthy food at time one (*r* = 0.41; *p* < 0.001), as well as conflict about healthy food at time one (*r* = 0.27; *p* < 0.001) and two (*r* = 0.45; *p* < 0.001). Children's BMI scores (t1) were positively related to conflict as well (*r* = 0.11; *p* < 0.05), so did food as a reward (t1; *r* = 0.24; *p* < 0.001). With regard to conflict about healthy food at time two, we found significant relationships with conflict about healthy food at time one (*r* = 0.54; *p* < 0.001) and conflict about unhealthy food at times one (*r* = 0.22; *p* < 0.001) and two (*r* = 0.45; *p* < 0.001). Familiar acceptance of healthy food (t1) negatively related to conflict about health food at time two (*r* = −0.19; *p* < 0.001). In addition, we observed a positive relationship of this conflict dimension with food as a reward (*r* = 0.19; *p* < 0.001). For an overview of all bivariate correlations, see Table 2.

**Table 2:** Bivariate correlations.

	**1**	**2**	**3**	**4**	**5**	**6**	**7**	**8**	**9**	**10**	**11**	**12**	**13**
1. Conflict about unhealthy food (t1)	1												
2. Conflict about unhealthy food (t2)	0.41^***^	1											
3. Conflict about healthy food (t1)	0.36^***^	0.27^***^	1										
4. Conflict about healthy food (t2)	0.22^***^	0.45^***^	0.54^***^	1									
5. Children's age (t1)	−0.01	0.01	−0.01	−0.02	1								
6. Children's zBMI scores (t1)	0.15^***^	0.11^*^	−0.03	−0.04	0.00	1							
7. Children's gender (t1)	0.03	−0.02	0.05	0.04	0.05	−0.00	1						
8. Familiar acceptance of healthy food (t1)	−0.19^***^	−0.08	−0.25^***^	−0.19^***^	0.06	0.06	0.03	1					
9. Active food-related mediation (t1)	−0.01	0.02	0.00	−0.03	0.02	−0.03	0.02	0.20^***^	1				
10. Restrictive food-related mediation (t1)	0.14^**^	0.06	0.04	0.11^*^	−0.03	−0.00	0.04	0.14^**^	0.42^***^	1			
11. Covert control (t1)	0.06	0.03	0.01	0.01	−0.01	0.08	0.02	0.10^*^	0.31^***^	0.39^***^	1		
12. Restriction of food access (t1)	0.13^**^	0.05	0.08	0.04	0.02	0.11^*^	0.05	0.08	0.27^***^	0.44^***^	0.41^***^	1	
13. Use of food as a reward (t1)	0.29^***^	0.24^***^	0.15^***^	0.19^***^	−0.02	0.04	0.05	−0.07	0.02	0.11^*^	0.06	0.15^***^	1

* *p < 0.05*;

** *p < 0.01*;

*** *p < 0.001*.

### Autoregressive Effect

Our regression models revealed that parent-child conflict about unhealthy food at the first wave positively predicted parent-child conflict about unhealthy food at the second wave (*b* = 0.34; ß = 0.31; *p* < 0.001). Also, parent-child conflict about healthy food of the first wave positively predicted parent-child conflict about healthy food of the second wave (*b* = 0.56; ß = 0.54; *p* < 0.001).

### Food-Related Mediation

The results of our regression analysis indicated no main effect of restrictive food-related mediation (t1) on parent-child conflict about unhealthy food (t2; *b* = 0.01; ß = 0.02; *p* = 0.738). However, the use of restrictive food-related mediation (t1) increased parent-child conflicts about healthy food (t2; *b* = 0.13; ß = 0.16; *p* = 0.001), hence only partly supporting H1 which assumed that parents' use of restrictive food-related mediation may lead to the emergence of parent-child conflict about both a) unhealthy and b)healthy food. Moreover, active food-related mediation (t1) did not significantly affect parent-child conflict about both unhealthy (t2; *b* = 0.00; ß = 0.00; *p* = 0.937) and healthy food (t2; *b* = −0.04; ß = −0.04; *p* = 0.313). Thus, H2 which assumed that active mediation can decrease conflict about (a) unhealthy and (b) healthy food did not find support.

### Feeding Practices

#### Overt Control

With regard to overt control, we found that the use of food as a reward (t1; *b* = 0.09; ß = 0.13; *p* = 0.002) but not the restriction of food access (t1; *b* = −0.02; ß = −0.04; *p* = 0.416) increased parent-child conflict about unhealthy food (t2). Our analyses also revealed an impact of the use of food as a reward over time (t1; *b* = 0.10; ß = 0.09; *p* = 0.015) but not of parents' restriction of food access (t1; *b* = −0.03; ß = −0.04; *p* = 0.313) in the case of conflicts about healthy food (t2). These results thus lend support to the assumption (H3) that food as a reward positively relates to an increase in conflict about a) unhealthy and b) healthy food. Yet, our hypothesis regarding restriction of food access increasing conflict about food (H4) is not supported.

#### Covert Control

Moreover, our analysis revealed no predictive impact of parental covert control (t1; *b* = 0.01; ß =0.01; *p* = 0.868) on parent-child conflict about unhealthy food (t2). Covert control (t1) also did not lead to higher potential for conflict between parents and their children about healthy food (t2; *b* = −0.03; ß = −0.03; *p* = 0.460). These findings lend no support to our assumptions that covert control increases parent-child food conflicts (H5). Table 3 summarizes the results for parent-child conflicts about *unhealthy food* while Table 4 includes findings for parent-child conflicts about *healthy food*.

**Table 3:** Multiple linear regression on parent-child conflicts about unhealthy food.

	**Dependent variable: parent-child conflict about unhealthy food (t2)**					
**Independent variables:**	**b**	**SE**	**ß**	***p*****-value**	***$R_{corr}^{2}$***	***ΔR**^**2**^*
Step 1: Autoregressive control					0.17	
Conflict about unhealthy food (t1)	**0.34**	**0.05**	**0.31**	**0.000**		
Step 2: Control and main variables					0.19	0.04
Conflict about healthy food (t1)	**0.11**	**0.03**	**0.16**	**0.000**		
Children's age (t1)	−0.03	0.03	−0.04	0.381		
Children's gender (t1)	−0.07	0.08	−0.04	0.348		
Children's zBMI scores^a^ (t1)	0.06	0.03	0.08	0.071		
Familiar acceptance of healthy food (t1)	0.02	0.03	0.03	0.544		
Active food-related mediation (t1)	0.00	0.03	0.00	0.937		
Restrictive food-related mediation (t1)	0.01	0.03	0.02	0.738		
Covert control (t1)	0.01	0.03	0.01	0.868		
Restriction of food access (t1)	−0.02	0.02	−0.04	0.416		
Use of food as a reward (t1)	**0.09**	**0.03**	**0.13**	**0.002**		
Step 3: Interaction effects					0.20	0.01
Active mediation * food as a reward	0.02	0.02	0.05	0.289		
Restrictive mediation * food as a reward	**0.04**	**0.02**	**0.09**	**0.029**		

*N = 501; 38 missing values*;

a *children's standard deviation scores of BMI. Bold values highlight significant effects (p < 0.05)*.

**Table 4:** Multiple linear regression on parent-child conflicts about healthy food.

	**Dependent variable: parent-child conflict about healthy food (t2)**					
**Independent variables**	**b**	**SE**	**ß**	***p*****-value**	***$R_{corr}^{2}$***	***ΔR**^**2**^*
Step 1: Autoregressive control					0.30	
Conflict about healthy food (t1)	**0.56**	**0.04**	**0.54**	**0.000**		
Step 2: Control and main variables					0.32	0.03
Conflict about unhealthy food (t1)	−0.09	0.07	−0.06	0.186		
Children's age (t1)	−0.03	0.04	−0.03	0.455		
Children's gender (t1)	0.10	0.10	0.04	0.346		
Children's zBMI scores^a^ (t1)	−0.01	0.05	−0.00	0.917		
Familiar acceptance of healthy food (t1)	−0.07	0.04	−0.06	0.100		
Active food-related mediation (t1)	−0.04	0.04	−0.04	0.313		
Restrictive food-related mediation (t1)	**0.13**	**0.04**	**0.16**	**0.001**		
Covert control (t1)	−0.03	0.04	−0.03	0.460		
Restriction of food access (t1)	−0.03	0.03	−0.04	0.313		
Use of food as a reward (t1)	**0.10**	**0.04**	**0.09**	**0.015**		
Step 3: Interaction effects					0.33	0.02
Active mediation * food as a reward	−0.02	0.03	−0.03	0.408		
Restrictive mediation * food as a reward	**0.08**	**0.02**	**0.13**	**0.001**		

*N = 502; 39 missing values*;

a *children's standard deviation scores of BMI. Bold values highlight significant effects (p < 0.05)*.

### Inconsistency of Food-Related Mediation Strategies and the Use of Food as a Reward

**Figure 2:**
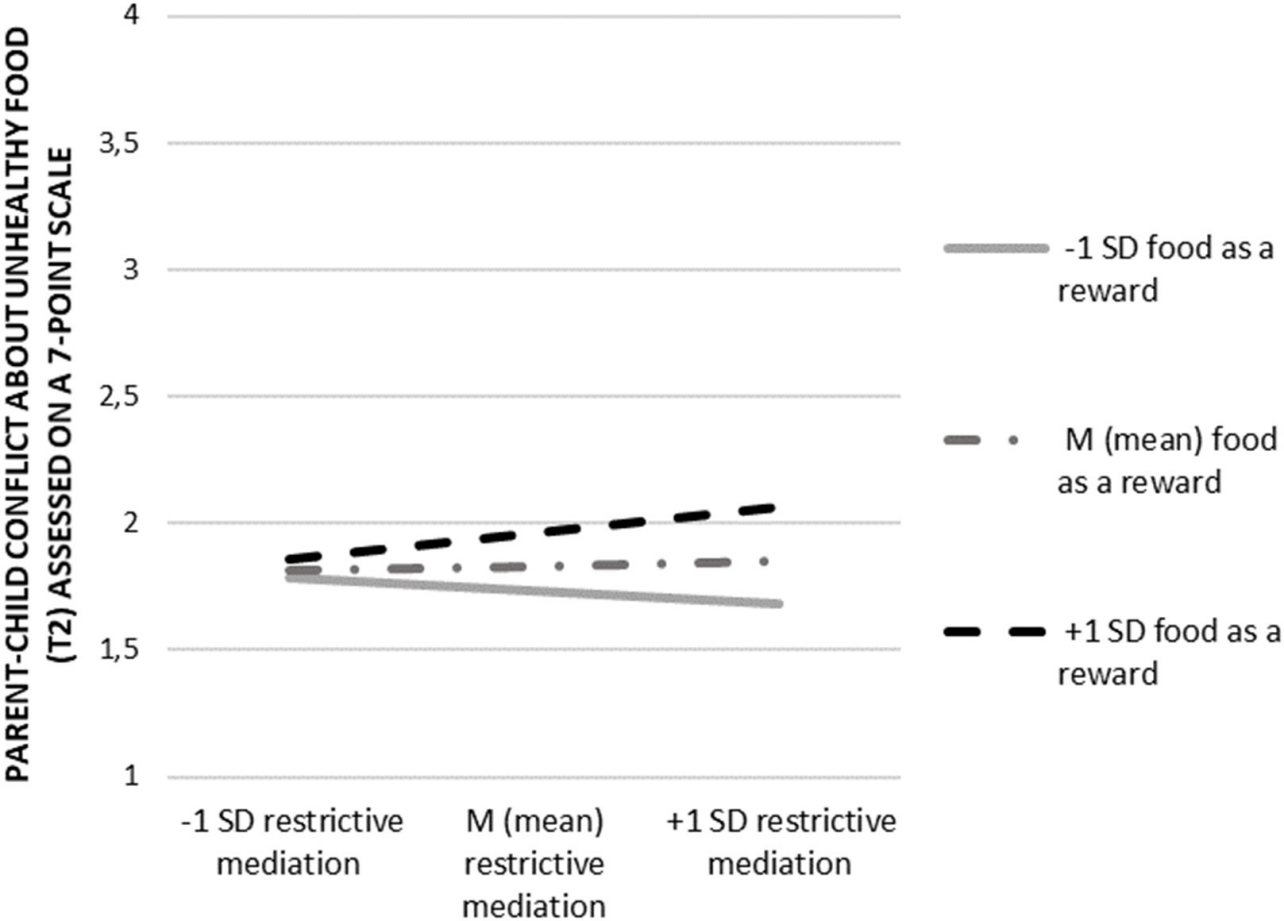
Interaction effect of restrictive food-related mediation (t1) and the use of food as a reward (t1) on parent-child conflict about unhealthy food (t2).

For the interplay between parents' food-related mediation strategies and their use of food as a reward (RQ1), we found a significant interaction effect of restrictive food-related mediation (t1) and the use of food as a reward (t1) on parent-child conflict about unhealthy food (t2; *b* = 0.04; ß = 0.09; *p* = 0.029; for a visualization of the interaction effect see Figure 2). As an examination of the interaction term indicated, above a medium level of restrictive food-related mediation (above the threshold of 3.20 assessed on a 7-point scale) using food as a reward often (+1SD), significantly increased the potential for conflict about unhealthy food (t2).

**Figure 3:**
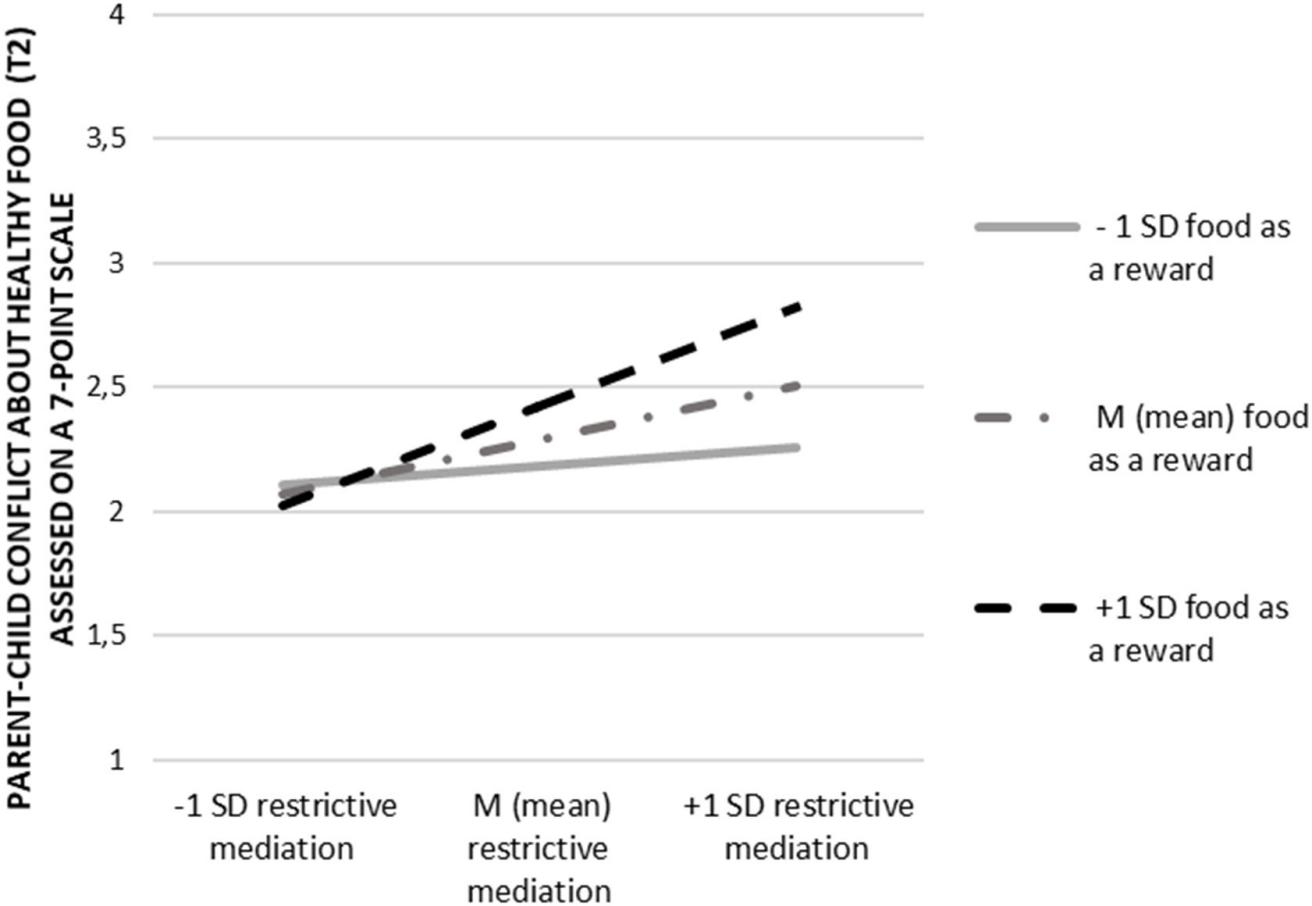
Interaction effect of restrictive food-related mediation (t1) and the use of food as a reward (t1) on parent-child conflict about healthy food (t2).

In line, the results of the second regression analysis also showed an interaction effect of restrictive food-related mediation (t1) and the use of food as a reward (t1) on parent-child conflict about healthy food (t2; *b* = 0.08; ß = 0.13; *p* = 0.001; for a visualization of the interaction effect see Figure 3). An examination of the interaction term indicated that at above a medium level of restrictive food-related mediation (above the threshold of 3.72 assessed on a 7-point scale), using food as a reward on occasion (M) or often (+1SD), significantly increased the potential for conflict about healthy food (t2).

In contrast, active food-related mediation (t1) and the use of food as a reward (t1) did not show an interaction effect for neither of the examined dependent variables [conflicts about unhealthy food (t2): *b* = 0.02; ß = 0.05; *p* = 0.289; conflicts about healthy food (t2): *b* = −0.02; ß = −0.03; *p* =0.408].

### Control Variables

Regarding our controls, we observed that parent-child conflicts about healthy food (t1) served as a positive indicator (*b* = 0.11; ß = 0.16; *p* < 0.001) for parent-child conflicts about unhealthy food (t2). There was also a marginal significant effect of children's BMI (t1) on parent-child conflict about unhealthy food (t2; *b* = 0.06; ß = 0.08; *p* = 0.071). However, children's age (t1), children's gender, as well as familiar acceptance of healthy food (t1) did not relate to this conflict (t2; see Table 3). With respect to healthy food conflicts (t2), neither parent-child conflicts about unhealthy food (t1) of the first wave nor one of the control variables (t1) related to this particular parent-child conflict (see Table 4).

## Discussion

Our study's aim was to gain insights into how conflicts about healthy and unhealthy food can be explained by parents' communication strategies and feeding practices. Results showcase that parents' use of unhealthy food as a reward at time one relates to both healthy and unhealthy food conflicts at time two. Furthermore, inconsistent parental educational styles increased the conflict potential about both unhealthy and healthy food. Active food-related mediation and covert control did not increase nor decrease food-related conflicts about unhealthy and healthy food.

With regard to the observed autoregressive effects in our statistical model, we found that while both autoregressive effects indicate a positive prediction of our conflict measures between t1 and t2, the autoregressive effect for conflict about unhealthy food was medium to small. This result suggests that the conflict measurement about unhealthy food is not as stable as the employed measurement of conflict about healthy food (50). Our results also showed that when conflicts about healthy food emerge this does not automatically mean that conflicts about unhealthy food will arise as well. While, our analysis revealed that conflicts about healthy food at time 1 served as a relevant indicator for conflicts about unhealthy food at time 2, we did not find the same relationship for conflicts about unhealthy food (t1) as an indicator for conflict abut healthy food (t2). This result implies that the conflict potential with regard to food in general may be higher in families in which conflicts about unhealthy food are present compared to families that are mainly characterized by conflicts about healthy nutrition.

With regard to the parents' communication strategies, we observed that neither active nor restrictive food-related mediation at time 1 positively related to the emergence of parent-child conflict about unhealthy food at time 2. In addition, active food related mediation did not show a negative relationship to the conflict about healthy food. Thus, active food related mediation is might not be a sufficient measure to decrease conflicts about food, even though it is based on a reflected and understanding-oriented type of communication (26, 28). However, in the case of conflicts about healthy food findings indicated an increase in conflict potential coinciding with an increased use of restrictive food-related mediation. In other words, parents that forbid their child to eat specific food products and provide a set of strict rules on what and how much their child is allowed to eat, might increase the likelihood of a parent-child conflict about healthy food in the immediate future. An explanation for this circumstance may be that healthy food options do not align with children's inherent preferences (22), leading to an increased likelihood that a parent-child conflict breaks out (4). Hence, if parents do not argue the benefits of healthy food conclusively and solely dictate their children what to eat by setting rules without any additional explanation, conflicts may arise. This might also explain the expected missing relationship of active food-related mediation on arising conflicts about food. The study of Gram supports these findings by concluding that children whose parents draw the ‘health card' through a health discourse may perceive health as an uncontested, important domain and thereby “…help both minimizing unhealthy food purchase and in avoiding conflicts.” [(30), p. 188]

With regard to parents' feeding practices, we found that particularly the use of food as a reward appears to have an important relationship with conflicts about food. Hence, parents frequent use of certain food as a reward in exchange for good behavior at time 1 coincides with conflict situations about food with their child at time 2. Existing research so far only gave indications to a potential relationship between this kind of feeding practice and food-related conflicts (2, 21, 24, 30, 41). Our results show for the first time that rewarding children with their favorite food can potentially contribute to the development of parent-child conflicts about food.

Yet, the restriction of food access did not lead to a higher likelihood of parent-child conflicts. The missing positive impact of parents' restriction of food access was somewhat surprising. Especially in the case of parent-child conflict about unhealthy food as our employed measurement specifically related to the restriction of access to unhealthy food. We suppose that our used items may also illustrate a more overall measurement than other questionnaires. For instance, the Restricted Access Questionnaire (RAQ) specifically assess the phenomena “keeping unhealthy foods out of reach” (through the use of items such as “At home, do you try to keep any of these foods out of your child's reach, so that your child cannot physically reach it?”) [(39), p.34].

With regard to explaining how emergence about parental-child conflict relates to covert control, we found no relationship. While previous studies have indicated that covert feeding practices might be helpful to steer children to more healthy food alternatives (21), controlling children's access to food in a non-observable manner does not have the potential to diminish conflicts.

Lastly, our study gives insights into the interplay between restrictive food-related mediation and the use of food as a reward (RQ1). More precisely, we found that a greater use of both restrictive food-related mediation and food as a reward related to slight increases in food conflicts. This finding is also in line with previous research which outlined that parents' increased use of restrictive feeding practices has shown to be counterproductive in connection with children's diet (36). This may also hold true for parent-child conflicts about food. Compared to this, active-food-related mediation and the use of food as a reward did not interact. Given this finding, we suppose that the parental use of active food-related mediation in combination with food as a reward may not be that commonly applied by parents. Indeed, while the use of food as a reward positively correlates with restrictive food-related mediation, additional analysis yielded no correlation with active food-related mediation (see Table 2).

Based on our findings, it might be also important to discover at what level of parent-child conflict about food is really harmful for children's well-being. For instance, although a joint purchase of food can be associated with family conflicts (4), involving children in meal planning and preparations as well as in grocery shopping is assumed to equip children with skills that are important for future nutritional decisions (51). We thus argue that food-related “conflicts” should not be completely avoided but are part of the familiar negotiation process that helps children in their developmental process. However, here we are rather referring to a “health discourse” which is closely related to the concept of active food-related mediation (26, 28). As our results show, this communication strategy does not predict food-related conflicts, which may be harmful for children's future diet.

The present study delivers several key findings that have significant implications for society and research. Most importantly, parents' use of restrictive and controlling patterns seems to potentially increase parent-child conflicts about food. Especially rewarding children with food for a desired behavior may seem intuitive to parents, but it appears to be counterproductive. In our study, the use of food as a reward positively related to the chance of food-related conflicts. In combination with a restrictive food-related mediation style we found that this main effect could be pushed even further. Yet, the observed interaction effects were notably, relatively small. Still following the observed results, parents on the one hand communicate strict rules of what to eat and not to eat while at the same time the communicated “forbidden” foods are then used to reward children. We call this phenomenon “the forbidden reward.” This obvious inconsistency seems to fuel conflict over time, which affects both conflicts about healthy and unhealthy eating.

## Limitations and Future Research

The present study is based on a rather large sample and provides both information on parents as well as their children. It also gives a longitudinal view on our research objective and therefore provides a new perspective on family conflicts about nutrition. Furthermore, it adds to a so far under-researched but important subject. Yet, our study also faces some limitations. Most importantly, due to the limited existing research in this area we used a self-created measurement for parent-child conflict about food. Although reliability values were acceptable for our two factors, and the factor loadings of our explorative factor analysis speak to the internal consistency of the measure, additional research is needed to further expand this instrument. More specifically, conflicts about food may also include other aspects than considered by our definition.

In addition, since the use of food as a reward has been frequently referenced in the context of parent-child conflict about food, we mainly focused on the interplay between parents' communication strategies and the use of food as a reward. Future research is encouraged to investigate the interplay with other relevant feeding practices that may also lack consistency when combining them with occurring communication strategies. We also suggest that future studies should include additional indicators of pre-existing eating behavior and food preference as we have only asses this through one factor in our analysis. In doing so, we are getting closer to fully predict how parent-child conflicts about food emerge.

Finally, since parental food-related mediation styles, unlike parental feeding practices, have hardly been explored, we suggest to expand the concepts used. Particularly in the case of active food-related mediation, the “aim” of the parental explanation might make a difference in terms of emerging conflicts [i.e., either promotion-focused or prevention-focused; Melbye and Hansen (52)]. Future studies should also consider the use of other empirical approaches, such as qualitative examinations or experimental designs combined with eye-tracking (53) to further examine how parental feeding and mediation practices correlate with conflict about food. The present study is not able to proof causality of the indicated relationships. Hence, future research should particularly focus on experimental research which examines the outcomes of different parental styles. Still, this study was able to give a first insight into the relationship of parental feeding and mediation practices and parent-child conflicts about food over the course of 6 months, which is a valuable addition to literature.

## Conclusion

In sum, our study revealed that parents can potentially escape triggering conflicts about food by using and avoiding certain patterns. Especially the discussion of the health benefits of certain food products and the consequences of unhealthy eating behavior may prevent the emergence of food-related conflicts or at least should not spark them. In addition to this, our findings support the point of view that covert feeding practices such as covert control might be more promising than overt, restrictive practices (36). With our study, we hope to spark the discussion about one of the most critical arenas for conflict between parents and children that lay the groundwork for a healthy nutritional behavior in the future.

## Data Availability Statement

The raw data supporting the conclusions of this article will be made available by the authors, without undue reservation.

## Ethics Statement

The studies involving human participants were reviewed and approved by Ethics Committee of the University of Vienna. Written informed consent to participate in this study was provided by the participants' legal guardian/next of kin.

## Author Contributions

IS conceptualized the study, the data collection instruments, collected data, finalized the analysis, and drafted the initial manuscript. BN conceptualized the data collection instruments, carried out the initial analysis, and reviewed and revised the manuscript. AB conceptualized the study, collected data, and reviewed and revised the manuscript. JM supervised study conceptualization, data collection instruments, data collection, as well as data analysis, and reviewed and revised the manuscript. All authors approved the final manuscript as submitted and agree to be accountable for all aspects of the work.

## Conflict of Interest

The authors declare that the research was conducted in the absence of any commercial or financial relationships that could be construed as a potential conflict of interest.

## Footnotes

^1^To exclude selective drop-out, we additionally searched for any conspicuous differences between the means and SDs for all variables of interest in the overall sample (*N* = 829) and the means and SDs in the finale sample (*N* = 541). No drop-out patterns were observed.
